# Patient-reported functional outcome measures and treatment choice for prostate cancer

**DOI:** 10.1186/s12894-022-01117-1

**Published:** 2022-11-05

**Authors:** Tenaw Tiruye, Michael O’Callaghan, Kim Moretti, Alex Jay, Braden Higgs, Kerry Santoro, Terry Boyle, Kerry Ettridge, Kerri Beckmann

**Affiliations:** 1 Cancer Epidemiology and Population Health Research Group, Allied Health and Human Performance, University of South Australia, Adelaide, South Australia; 2 South Australian Prostate Cancer Clinical Outcomes Collaborative, Adelaide, South Australia; 3 Flinders Health and Medical Research Institute, Flinders University, Adelaide, South Australia; 4 Discipline of Medicine, University of Adelaide, Adelaide, South Australia; 5 Discipline of Surgery, University of Adelaide, Adelaide, South Australia; 6 Flinders Medical Centre, Bedford Park, South Australia; 7 Department of Radiation Oncology, Royal Adelaide Hospital, Adelaide, South Australia; 8 Urology Unit, Southern Adelaide Local Health Network, Adelaide, South Australia; 9 Australian Centre for Precision Health, University of South Australia, Adelaide, South Australia; 10 Health Policy Centre, South Australian Health and Medical Research Institute, Adelaide, South Australia; 11 School of Public Health, University of Adelaide, Adelaide, South Australia; 12 Public Health Department,, Debre Markos University,, Debre Markos, Ethiopia

**Keywords:** Prostate cancer, Patient reported outcome measure, Quality of life, Australia

## Abstract

**Background:**

The aim of this study was to describe changes in patient-reported functional outcome measures (PROMs) comparing pre-treatment and 12 months after radical prostatectomy (RP), external beam radiation therapy (EBRT), brachytherapy and active surveillance (AS).

**Methods:**

Men enrolled from 2010 to 2019 in the South Australian Prostate Cancer Clinical Outcomes Collaborative registry a prospective clinical registry were studied. Urinary, bowel, and sexual functions were measured using Expanded Prostate Cancer Index Composite (EPIC-26) at baseline and 12 months post-treatment. Higher scores on the EPIC-26 indicate better function. Multivariable regression models were applied to compare differences in function and extent of bother by treatment.

**Results:**

Of the 4926 eligible men, 57.0% underwent RP, 20.5% EBRT, 7.0% brachytherapy and 15.5% AS. While baseline urinary and bowel function varied little across treatment groups, sexual function differed greatly (adjusted mean scores: RP = 56.3, EBRT = 45.8, brachytherapy = 61.4, AS = 52.8; p < 0.001). Post-treatment urinary continence and sexual function declined in all treatment groups, with the greatest decline for sexual function after RP (adjusted mean score change − 28.9). After adjustment for baseline differences, post-treatment sexual function scores after EBRT (6.4; 95%CI, 0.9–12.0) and brachytherapy (17.4; 95%CI, 9.4–25.5) were higher than after RP. Likewise, urinary continence after EBRT (13.6; 95%CI, 9.0-18.2), brachytherapy (10.6; 95%CI, 3.9–17.3) and AS (10.6; 95%CI, 5.9–15.3) were higher than after RP. Conversely, EBRT was associated with lower bowel function (− 7.9; 95%CI, − 12.4 to − 3.5) than RP. EBRT and AS were associated with lower odds of sexual bother (OR 0.51; 95%CI, 0.29–0.89 and OR 0.60; 95%CI, 0.38–0.96, respectively), and EBRT with higher odds of bowel bother (OR 2.01; 95%CI, 1.23–3.29) compared with RP.

**Conclusion:**

The four common treatment approaches for prostate cancer were associated with different patterns of patient-reported functional outcomes, both pre- and 12 months post-treatment. However, after adjustment, RP was associated with a greater decline in urinary continence and sexual function than other treatments. This study underscores the importance of collecting baseline PROMs to interpret post-treatment functional outcomes.

**Supplementary Information:**

The online version contains supplementary material available at 10.1186/s12894-022-01117-1.

## Background

Prostate cancer survival is high in developed countries due to the availability of prostate specific antigen testing and effective treatment options. The five-year relative survival for men with prostate cancer in Australia is approximately 96% [1]. Given the high probability of long-term survival from prostate cancer, maintaining quality of life should also be central to clinical practice [2]. However, men diagnosed with prostate cancer are faced with challenging decisions about treatment choices, due to the substantial risk of adverse effects with most recommended treatments. These can include post-treatment impact on urinary, bowel and sexual functioning [3–8]. Understanding the negative effects of the treatments and how the treatments affect quality of life of men has become crucial for decision making [6].

It is increasingly recognised that selecting appropriate treatments needs to be tailored to each man’s circumstances to minimize impacts on physical functioning and mental wellbeing. To this end, greater emphasis has been placed on collecting information from patients about their physical functioning (i.e., patient-reported outcome measures (PROMs)) to audit and evaluate various treatment outcomes [2]. Because they are derived from the patient’s perspective, self-reported measures of functional outcomes such as erectile function and urinary continence are very important [9]. Functional outcomes are often under-reported by physicians [10] and PROMs can guide clinical practice to be more responsive to individual patients’ needs and inform ways in which patients can self-manage their condition [2].

The Prostate Cancer Outcomes Registry Australia and New Zealand (PCOR-ANZ), which was established primarily to monitor and improve outcomes for men with prostate cancer [11], collects and reports post-treatment PROMs data. However, this collection does not include baseline PROMs, due to logistical difficulties identifying men before they commenced treatment. Baseline PROMs assessment is important for interpreting post-treatment impacts, given the differing age and comorbidity profiles among those who undergo different treatment options. In addition, baseline PROMs are useful in identifying patients at risk of impaired function, to facilitate treatment decision-making by patients and clinicians [12] and to avoid patients’ regret due to unexpected physical outcomes of treatment [13].

Currently, Australian data on baseline functioning among men with prostate cancer are sparse. However, the long-standing South Australian Prostate Cancer Clinical Outcomes Collaborative (SA-PCCOC) database (which contributes core data to PCOR-ANZ) has collected PROMs data at baseline and sequential time points after diagnosis from enrolled participants over the past two decades. The aim of this study was to describe PROMs at baseline (pre-treatment) and after 12 months of follow-up, and to compare the extent to which functional outcomes are impacted by four common prostate cancer treatments— radical prostatectomy (RP), external beam radiation therapy (EBRT), brachytherapy and active surveillance—using PROMs data collected by SA-PCCOC.

## Methods

### Data source

Data were accessed from SA-PCCOC registry [14]. SA-PCCOC is a disease-specific, multi-institutional, prospective registry which has been operating since 1998. Since 2008, the registry has been expanded to collect clinical and oncologic data from both public and private treatment centres and captures data from approximately 90% of men with prostate cancer in South Australia. While intervals for collecting PROMs have changed over time, baseline and 12 months PROMs have been consistently collected since the registry’s inception.

### Sampling

**Fig. 1:**
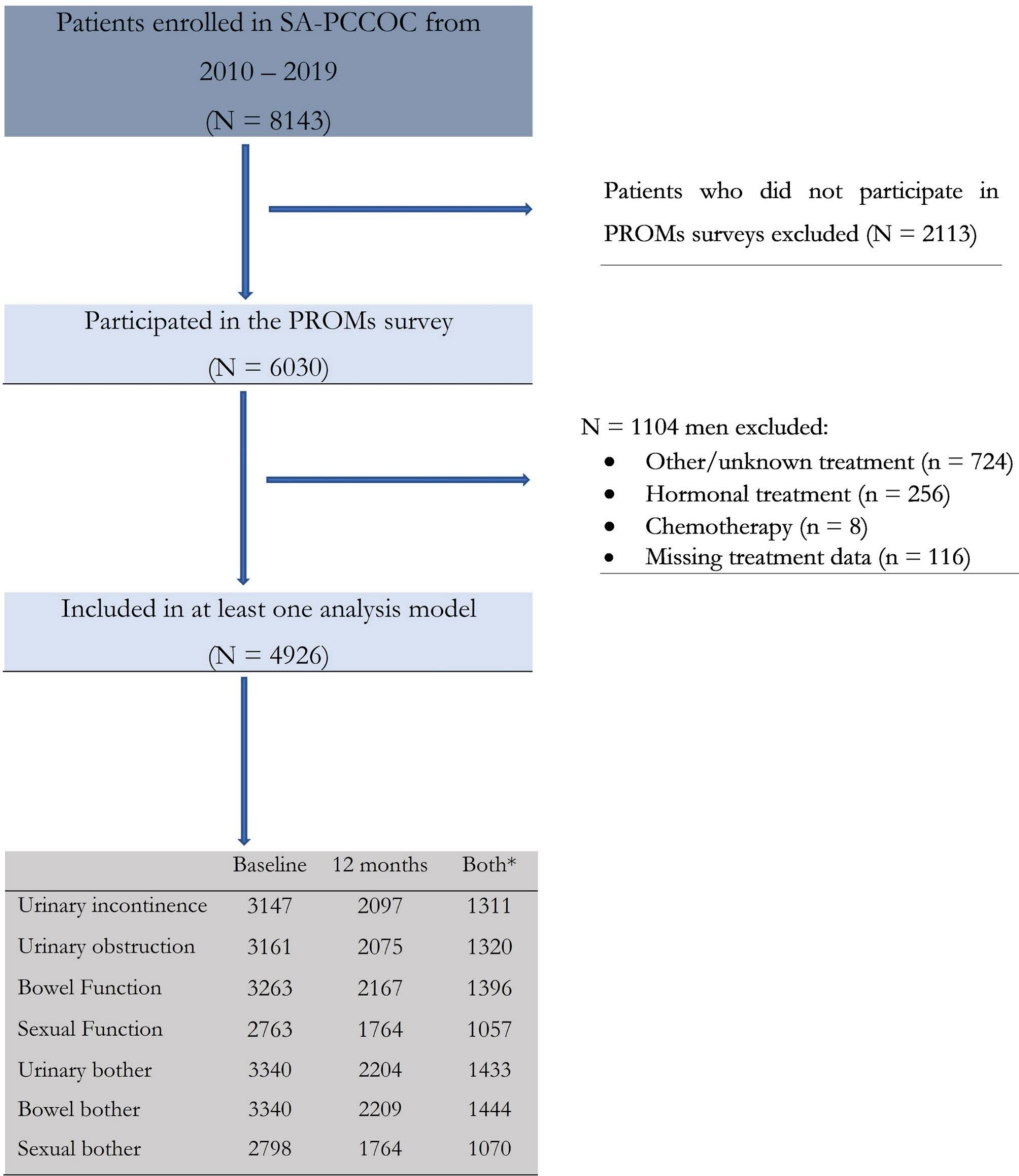
Selection procedure of study participants *Number of men who completed both baseline and 12 months patient reported functional outcome measures

This study included men enrolled in SA-PCCOC who were diagnosed between 2010 and 2019 and had completed any items in the PROMS surveys at baseline or 12 months post treatment. This period was selected to (1) reflect contemporary treatments practices, (2) approximate complete population-based coverage, and (3) be inclusive of both public and private patients. In total, 8143 men were enrolled in SA-PCCOC from 2010 to 2019, of whom 6030 had participated baseline and/or 12-month PROMs surveys. Those who received RP, EBRT, brachytherapy or active surveillance were included in analyses (n = 4926). Men undergoing primary hormonal treatment (n = 256), chemotherapy (n = 8), other/unknown treatment (n = 724) or with missing treatment data (n = 116) were excluded (Fig. 1).

### Measurement and variables

#### Outcomes

The first set of outcome variables were the functional outcome scores: at baseline (pre-treatment) and 12 months after treatment commenced. Functional outcomes were measured using Expanded Prostate Cancer Index Composite (EPIC)-26 domain scores [15]. Four items were used in the urinary incontinence domain, four items for urinary irritative/obstructive symptoms, six items for bowel function, and six items for sexual function. Each functional domain was calculated in accordance with previous EPIC-26 guidelines [16]. Accordingly, if $\ge$20% of the items in a domain were missing a response, that domain was not calculated. Domain scores range from 0 to 100 with a higher score indicating better function. An established convention of minimal clinically important difference (MCID) in EPIC-26 domain score of PROMs [17] was used to describe and complement the reported scores. MCIDs were set at 10-point score differences for sexual function, 6-point for urinary incontinence, 5-point for urinary obstruction, and 4-point for bowel function.

The second set of outcome variables were extent of “bother” regarding urinary, bowel and sexual function. Each bother outcome was measured using a single item question from the EPIC-26 that asked men how big a problem their [urinary symptoms, bowel habits, lack of sexual function] had been for them in the last four weeks. The responses were: no signs, very small, small, moderate, or big bothers. For descriptive reporting, we merged “no signs”, “very small” and “small” together and “moderate” with “big” bother but retained the original five responses in the final multivariable analyses.

#### Treatments

Primary treatments were grouped as RP, EBRT, brachytherapy and active surveillance based on predefined criteria used by SA-PCCOC. Men were categorised as having active surveillance if this was recorded as their initial treatment in their medical record or derived through registry algorithms based on risk level and clinical characteristics. Between 2010 and 2016, active surveillance protocol would include a repeat ultrasound guided transrectal or transperineal biopsy by 12 months, with no routine use of magnetic resonance imaging (MRI) for surveillance. From 2017, MRI+/- transperineal biopsy became the more common practice. Adjuvant and salvage therapies were not accounted for due to inconsistent collection of secondary treatment data in SA-PCCOC registry.

#### Covariates

Sociodemographic characteristics included age at diagnosis (below 60, 60–64, 65–69, above 70) and socioeconomic status based on the 2016 Socio-Economic Indexes for Areas (SEIFA) score. SEIFA is an indicator of relative economic and social advantage/disadvantage of areas based on a range of socio-economic factors such as employment status, income and household status [18]. SEIFA scores were grouped into quintiles (lowest, low, medium, high, and highest socio-economic status). Risk classification was according to 2017 National Comprehensive Cancer Network (NCCN) groupings [19] and was derived from prostate-specific antigen (PSA) level, Gleason grade group and clinical stage of the disease; high risk (clinical stage T3a OR PSA > 20 ng/mL OR Gleason score of 8 or more), intermediate risk (clinical stage T2b and T2c OR PSA 10–20 ng/mL OR Gleason score of 7 (3 + 4 or 4 + 3)), and low risk (clinical stage T2a or earlier AND PSA < 10 ng/mL AND a Gleason score of less than 7). Diagnostic PSA (< 4, 4–10, 10–20 and > 20 ng/mL) and total Gleason score ( < = 6, 3 + 4, 4 + 3, 8 and 9–10) were coded as previously reported [11].

### Statistical analysis

Patient characteristics were summarized using descriptive statistics (frequency counts with percentages for categorical variables, means and standard deviations for normally distributed continuous variables, and median and interquartile range for continuous variables that were not normally distributed).

Functional outcomes were missing in a high proportion of men enrolled in SA-PCCOC, due to some surveys not being sent within specified time periods, survey non-response, or variability in response rates to specific EPIC-26 items. To derive population-wide estimates of functional scores we applied inverse probability weighting to all analyses. Weights were calculated by deriving propensity scores for ‘being a respondent’ for each specific functional outcome, using logistic regression to predict likelihood (propensity) of being a respondent within the entire SA-PCCOC cohort based on patient characteristics (with weight = 1/propensity score). The use of inverse probability weighting to account for missingness has been suggested in previous literature [20].

Multivariable linear regression models were fitted to estimate the effect of EBRT, brachytherapy, and active surveillance vs. RP on the 12 months functional outcome scores. Multivariate ordinal logistic regression models were fitted to identify the association between treatment types and patient-reported bother at 12 months. RP, which is the largest category, was considered as a reference group. Additional models were run with active surveillance as the reference group. Separate analyses were undertaken for each of the functional outcomes and bother items, with each model including only those participants with complete data for that specific outcome (number provided in Fig. 1). All the models were adjusted for covariates (age, SEIFA score, PSA level, risk category and primary symptoms) and respective baseline functional measures. Collinearity was checked using variance inflation factor (VIF) leading to “total Gleason score” being removed from the final adjusted models in favour of “risk category”. The final models present the mean difference in functional scores at 12 months between treatments after adjustment for baseline scores and other covariates, and adjusted odds ratio (aOR) for greater bother at 12 months, along with confidences intervals (CI) and *p-*values. Adjusted mean values of the outcome variables in each treatment category were calculated from postestimation predictions following multivariable regression models. Statistical significance was regarded as two-tailed $p<0.05$. All analyses were carried out using Stata version 15.0 (StataCorp, College Station, Tx, USA).

## Results

### Characteristics of study participants

A total of 4926 men were included in the analyses; mean age (sd), 66.1 (7.7) years; 43.5% had intermediate and 32.3% had high-risk disease. Regarding treatment, 2810 (57.0%) underwent RP, 1011 (20.5%) EBRT, 343 (7.0%) brachytherapy and 762 (15.5%) active surveillance. Men in the EBRT group were more likely to be older (mean age, 71.9 years vs. 64.5 years, p < 0.001) and have high-risk disease (57.2% vs. 30.8%, p < 0.001) than men in the RP group. Most men on active surveillance had low risk disease (n = 581, 76.2%) (Table 1).

**Table 1:** Sociodemographic and clinical characteristics of respondents, SA-PCCOC registry (2010–2019)

Variable	Treatment type								
	RP (n = 2810)		EBRT (n = 1011)		Brachytherapy (n = 343)		AS (n = 762)		p-value
	No.	%	No.	%	No.	%	No.	%	
Age, mean (sd)	64.5 (6.9)		71.9 (7.2)		65.7 (7.0)		64.8 (7.7)		
Below 60	636	22.6	52	5.1	70	20.4	202	26.5	< 0.001
60–64	639	22.7	110	10.9	70	20.4	135	17.7	
65–70	969	34.5	229	22.7	113	32.9	247	32.4	
Above 70	566	20.1	620	61.3	90	26.2	178	23.4	
Risk category									
Low	423	15.1	67	6.6	66	19.2	581	76.2	< 0.001
Intermediate	1442	51.3	339	33.5	185	53.9	77	10.1	
High	865	30.8	578	57.2	66	19.2	8	1.0	
Missing	80	2.8	27	2.7	26	7.6	96	12.6	
SEIFA									
Lowest	494	17.6	260	25.7	81	23.6	145	19.0	< 0.001
Low	434	15.4	214	21.2	71	20.7	139	18.2	
Medium	473	16.8	183	18.1	68	19.8	120	15.7	
High	668	23.8	180	17.8	72	21.0	196	25.7	
Highest	511	18.2	92	9.1	33	9.6	94	12.3	
Missing	230	8.2	82	8.1	18	5.2	68	8.9	
Diagnostic PSA level (ng/mL)									
<4	287	10.2	56	5.5	31	9.0	160	21.0	< 0.001
4–10	1705	60.7	388	38.4	214	62.4	461	60.5	
10–20	463	16.5	266	26.3	37	10.8	46	6.0	
>20	98	3.5	163	16.1	6	1.7	5	0.7	
Missing	257	9.1	138	13.6	55	16.0	90	11.8	
Gleason score									
<=6	594	21.1	112	11.1	93	27.1	714	93.7	< 0.001
3 + 4	1086	38.6	285	28.2	140	40.8	32	4.2	
4 + 3	604	21.5	206	20.4	69	20.1	1	0.1	
8	299	10.6	184	18.2	26	7.6	0	0.0	
9–10	204	7.3	204	20.2	10	2.9	0	0.0	
Missing	23	0.8	20	2.0	5	1.5	15	2.0	
Primary symptoms^**#**^									
Elevated PSA	2316	82.4	779	77.1	278	81.0	533	69.9	< 0.001
LUTS	76	2.7	78	7.7	8	2.3	96	12.6	
Other	52	1.9	25	2.5	9	2.6	28	3.7	
Missing	366	13.0	129	12.7	48	14.0	105	13.8	

RP, radical prostatectomy; EBRT, external beam radiation therapy; AS, active surveillance

sd, standard deviation; SEIFA, socio-economic indexes for areas; PSA, prostate-specific antigen; LUTS, lower urinary tract symptoms

^**#**^Erectile dysfunction (n = 3) was re-grouped under other

Adjusted mean functional domain scores and adjusted percentages of moderate-to-big bothers are described in the following sections. Crude data are presented in the supplementary tables.

### Sexual function

**Fig. 2:**
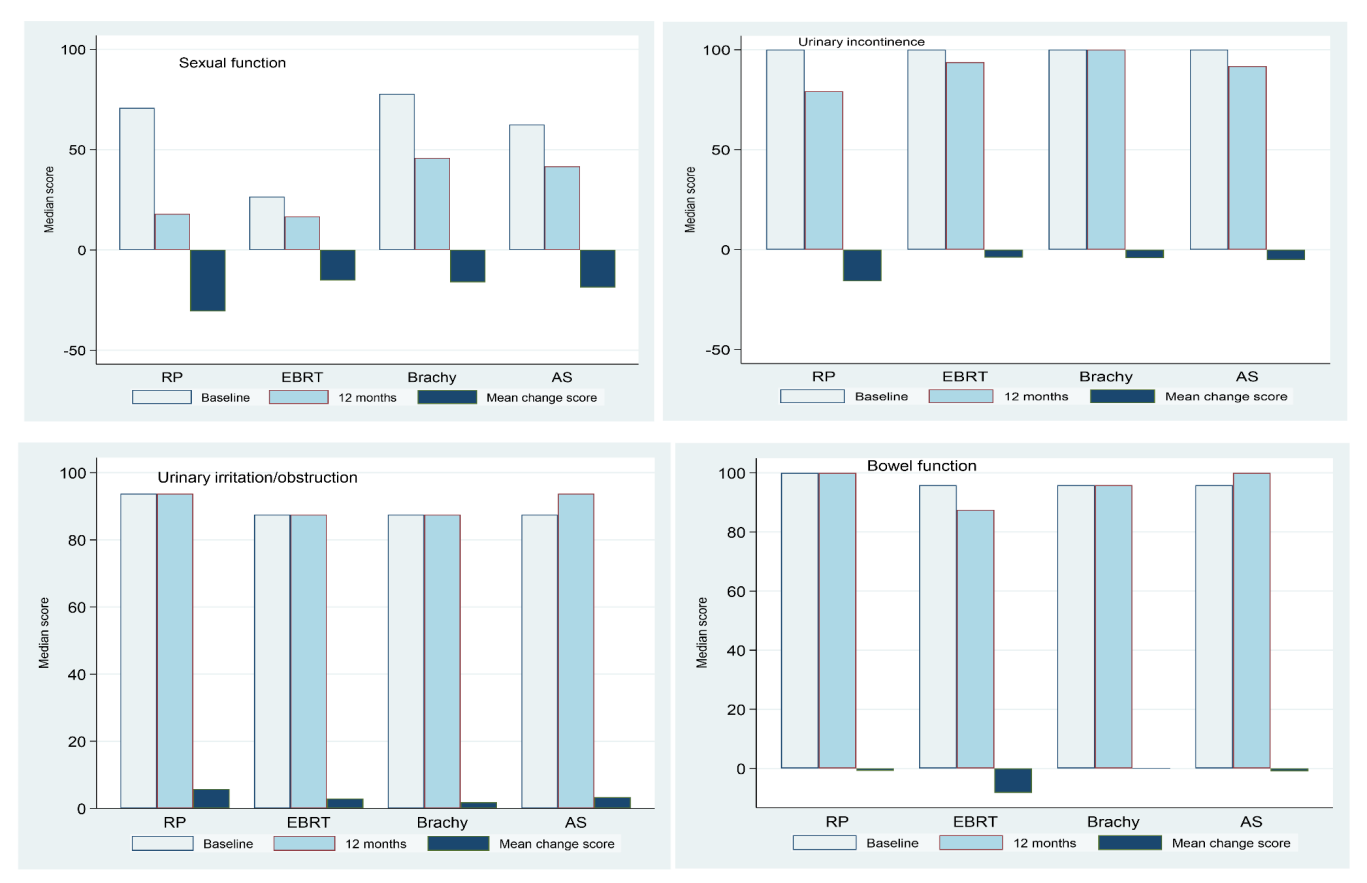
Patient reported functional outcome measures by treatment type RP, radical prostatectomy; EBRT, external beam radiation therapy; Brachy, brachytherapy; AS, active surveillance The y-axis denotes the unadjusted (crude) EPIC-26 domain scores, range from 0 (worst) to 100 (best). The baseline and 12 months EPIC-26 scores are in median score while the change scores are in mean score Negative change scores denote decline in function while positive change scores indicate improvement

At baseline (pre-treatment), men who underwent EBRT had lower baseline sexual function (adjusted mean score, 45.8) than men who underwent RP (adjusted mean score, 56.3), brachytherapy (adjusted mean score, 77.8) and active surveillance (adjusted mean score, 62.5). Sexual function domain scores had declined in all four treatment categories after 12 months, with lowest scores for men undergoing RP and EBRT (adjusted mean score, 28.5 and 35.0 respectively) (Table 2). Declines were clinically relevant (score change of > 10 points) in all the four treatment categories, with adjusted mean change score ranging from − 16.5 for EBRT to − 28.9 for RP (Table 2, Supplementary Table S1). Figure 2 and Supplementary Table S2 show unadjusted (crude) functional outcome scores across each treatment type.

**Table 2:** Adjusted mean functional outcome scores by treatment type, SA-PCCOC registry (2010–2019)

	Rx	Sexual function		Urinary incontinence		Urinary obstruction		Bowel function	
		Mean	95% CI	Mean	95% CI	Mean	95% CI	Mean	95% CI
Baseline scores‡	RP	56.3	54.1, 58.4	89.9	88.8, 91.0	85.9	84.8, 87.0	93.5	92.8, 94.3
	EBRT	45.8	41.7, 49.9	89.5	87.3, 91.6	82.2	79.9, 84.4	90.6	89.0, 92.2
	BT	61.4	54.6, 68.2	90.0	86.8, 93.2	83.1	79.7, 86.4	91.0	88.5, 93.5
	AS	52.8	48.4, 57.2	88.8	86.5, 91.1	84.1	81.7, 86.4	92.3	90.6, 94.1
12-month scores^¥^	RP	28.5	26.5, 30.6	74.4	72.5, 76.3	91.8	90.7, 92.8	92.7	91.7, 93.6
	EBRT	35.0	30.0, 39.9	88.0	84.0, 91.9	84.5	81.2, 87.8	84.7	80.5, 89.0
	BT	45.9	38.3, 53.6	85.0	78.4, 91.6	84.8	80.2, 89.4	90.8	87.6, 94.1
	AS	35.4	28.7, 42.2	85.0	80.8, 89.2	91.6	88.7, 94.4	90.6	88.4, 92.8
Change scores‡	RP	-28.9	-31.7, -26.2	-15.6	-17.7, -13.5	5.6	4.2, 6.9	-0.8	-1.8, 0.1
	EBRT	-16.5	-24.2, -8.8	-2.2	-6.5, 2.1	2.0	-2.7, 6.7	-6.3	-11.3, -1.2
	BT	-17.8	-27.1, -8.6	-5.4	-11.8, 1.1	1.7	-4.5, 7.8	-1.1	-4.9, 2.7
	AS	-22.1	-30.0, -14.3	-5.9	-10.2, -1.7	3.9	0.7, 7.0	-4.4	-6.6, -2.1
Mean difference^¥¥^	RP	reference							
	EBRT	6.4	0.9, 12.0*	13.6	9.0, 18.2***	-7.2	–10.7, − 3.8***	–7.9	–12.4, − 3.5***
	BT	17.4	9.4, 25.5***	10.6	3.9, 17.3***	-6.9	–11.7, − 2.2***	–1.8	–5.1, 1.5
	AS	6.9	–0.5, 14.3	10.6	5.9, 15.3***	-0.2	–3.5, 3.1	–2.1	–4.4, 0.2

Rx, treatment; RP, radical prostatectomy; EBRT, external beam radiation therapy; BT, brachytherapy; AS, active surveillance; CI, confidence interval

‡Adjusted for age, SEIFA score, PSA level, risk category and primary symptoms

^¥^Adjusted for baseline score of the respective function, age, SEIFA score, PSA level, risk category and primary symptoms

^¥¥^The adjusted mean difference in the 12 months functional scores between EBRT, brachytherapy, active surveillance vs. RP from linear regression models

All analyses were with the weighted sample

*p < 0.05, **p < 0.01, ***p < 0.001

In the adjusted linear regression model, compared with RP, the 12-month sexual function score was higher for patients who received EBRT (mean difference = 6.4 points; 95% CI, 0.9–12.0) and brachytherapy (mean difference = 17.4 points; 95% CI, 9.4–25.5) (Table 2). The 12-month post-treatment sexual function scores were higher for men undergoing brachytherapy (mean difference = 10.5 points; 95% CI, 0.3–20.7) compared with men on active surveillance (Supplementary Table S3).

The proportion of men reporting moderate-to-big sexual bother at baseline was relatively similar between EBRT (22.6%), RP (22.0%), brachytherapy (20.0%) and active surveillance (19.8%). Sexual bother was higher at 12 months than at baseline across all four treatment categories but was greatest among men who underwent RP (increased to 42.8% at 12 months from 22.0% at baseline) (Table 3). Supplementary Table S4 displays unadjusted (crude) proportions across all five categories of sexual bother (no sign, very small, small, moderate and big) at baseline and 12 months by treatment category. Results of the adjusted ordinal logistic regression showed men who received EBRT had about 50% lower odds of being in a higher category of sexual bother than those who underwent RP (aOR 0.51; 95% CI, 0.29–0.89) while those on active surveillance had about 40% lower odds (aOR 0.60; 95% CI, 0.38–0.96) (Table 4).

**Table 3:** Proportions of men who reported ‘moderate-to-big bothers’ by treatment type, SA-PCCOC registry (2010–2019)

Bother type	Rx	Baseline bother^#^		12 months bother^##^	
		**%**	**95% CI**	**%**	**95% CI**
Sexual bother	RP	22.0	19.3, 24.7	42.8	38.9, 46.8
	EBRT	22.6	16.9, 28.4	28.8	16.8, 40.8
	BT	20.0	10.8, 29.1	31.3	16.6, 45.9
	AS	19.8	14.4, 25.2	37.0	26.1, 47.8
Urinary bother	RP	9.7	7.9, 11.5	9.0	6.8, 11.2
	EBRT	13.6	9.6, 17.5	5.9	2.0, 9.9
	BT	11.6	5.1, 18.1	10.8	1.3, 20.3
	AS	12.1	7.3, 16.8	7.4	0.4, 14.3
Bowel bother^¥^	RP	3.5	2.4, 4.7	5.6	3.9, 7.3
	EBRT	6.1	3.1, 9.0	8.2	3.2, 13.2
	BT	4.5	0.6, 8.5	0	-
	AS	5.4	1.1, 9.7	10.4	2.0, 18.8

Rx, treatment type; RP, radical prostatectomy; EBRT, external beam radiation therapy; BT, brachytherapy; AS, active surveillance; CI, confidence interval

^#^Adjusted for age, SEIFA score, PSA level, risk category and primary symptoms

^##^Adjusted for baseline bother of the respective function, age, SEIFA score, PSA level, risk category and primary symptoms

Proportions were predicted from binary logistic regression models (moderate/big bother = 1 vs. no/very small/small bother = 0)

**Table 4:** Functional bothers and their association with treatment type, SA-PCCOC registry (2010–2019)

Rx	Sexual bother			Urinary bother			Bowel bother^¥^	
	**aOR‡**	**95% CI**		**aOR‡**	**95% CI**		**aOR‡**	**95% CI**
RP	reference			reference			reference	
EBRT	0.51	0.29, 0.89*		0.75	0.49, 1.15		2.01	1.23, 3.29**
BT	0.68	0.36, 1.27		0.76	0.41, 1.41		-	-
AS	0.60	0.38, 0.96*		0.64	0.40, 1.03		1.16	0.67, 2.02

Rx, treatment type; RP, radical prostatectomy; EBRT, external beam radiation therapy; BT, brachytherapy; AS, active surveillance; CI, confidence interval

^‡^The models were adjusted for inverse probability weight, baseline bother of the respective function, age, SEIFA score, PSA level, risk category and primary symptoms

All analyses were with the weighted sample

aOR, adjusted odds ratio from ordinal logistic regression models of 12-month post-treatment bothers (five categories: no, very small, small, moderate and big bothers)

^¥^The brachytherapy group was not included in the model because there were very small counts in brachytherapy group

*p < 0.05, **p < 0.01, ***p < 0.001

### Urinary function

#### Urinary incontinence

Baseline urinary incontinence domain scores were similar across the four treatment types with adjusted mean score ≈ 90 points. Urinary incontinence scores had declined in all four treatment categories after 12 months but the decline was clinically relevant only for men who had undergone RP (adjusted mean change score = − 15.6) (Table 2). In the final adjusted model, post-treatment urinary incontinence scores were higher for patients who received EBRT (mean difference = 13.6 points; 95% CI, 9.0-18.2), brachytherapy (mean difference = 10.6 points; 95% CI, 3.9–17.3), and active surveillance (mean difference = 10.6 points; 95% CI, 5.9–15.3) than men who had undergone RP (Table 2). There was no significant difference in urinary function when EBRT and brachytherapy were compared with active surveillance (Supplementary Table S3).

#### Urinary obstructive/irritative symptoms

Men in RP group had better baseline scores for urinary obstructive symptoms (adjusted mean score, 85.9) compared with men in the other three treatment groups. Urinary obstruction scores positively improved across all the four treatment types at 12 months, with a clinically significant improvement (adjusted mean change score, 5.6) among men who underwent RP (Table 2). In adjusted models, urinary obstruction scores at 12 months were lower for men undergoing EBRT (mean difference = − 7.2 points; 95% CI, − 10.7 to − 3.8) and brachytherapy (mean difference = − 6.9 points; 95% CI, − 11.7 to − 2.2) than those undergoing RP (Table 2). Compared with active surveillance, scores for 12-month post-treatment urinary obstruction were lower for men undergoing EBRT (mean difference = − 7.0 points; 95% CI, − 11.6 to − 2.5) and brachytherapy (mean difference = − 6.8 points; 95% CI, − 12.2 to − 1.3) (Supplementary Table S3).

#### Urinary bother

The proportion of men reporting baseline urinary bother was higher for EBRT than other treatment categories (moderate-to-big bother = 13.6%). The proportion of men reporting urinary bother had reduced slightly at 12 months in all treatment categories. There was no significant difference in post-treatment urinary bother between the treatments in the adjusted ordinal logistic regression model (Table 4).

### Bowel function

Men in RP group had slightly higher baseline median scores for bowel function compared with men who underwent radiotherapy (EBRT or brachytherapy). Likewise, at 12 months post-treatment, men who underwent RP had better bowel functional scores (adjusted mean score, 92.7 vs. 84.7 for EBRT). Men in EBRT and active surveillance groups had clinically meaningful decline in the bowel function after 12 months (adjusted mean change score, − 6.3 and − 4.4 respectively) (Table 2). In the final adjusted linear regression models, EBRT was associated with lower post-treatment bowel function than RP (mean difference = − 7.9 points; 95% CI, − 12.4 to − 3.5) (Table 2) and active surveillance (mean difference = − 5.8 points; 95% CI, − 10.7 to − 1.0) (Supplementary Table S3).

The proportion of men reporting baseline bowel bother was higher for EBRT than other treatment categories (6.1% moderate-to-big bother). Post-treatment bowel bother had increased in all treatment categories except in the brachytherapy group. In the adjusted ordinal logistic regression model, EBRT was associated with twice the odds of being in a higher bowel-related bother category than RP (aOR 2.01; 95% CI, 1.23–3.29). The brachytherapy group was excluded from the model due to no men in the brachytherapy group reporting big bowel bother (Table 4).

### Baseline vs. 12 months function

In the linear adjusted models, all baseline functional domains were positively associated with their respective 12 months domain scores. A one-point score increase in baseline urinary incontinence domain score was associated with a 0.45-point score (95% CI, 0.35–0.54) increase in urinary incontinence domain score after 12 months. Likewise, every one-point increase in baseline sexual function domain score was associated with 0.40 points higher score at 12 months (95% CI, 0.33–0.47).

## Discussion

Comparing the adverse side effects of treatment options for prostate cancer is a high research priority. In this study, patient-reported functional outcomes and related bother at baseline and at 12 months post-treatment are described for a contemporary Australian cohort and functional outcomes at 12 months compared across different treatment options after accounting for baseline function, clinical and sociodemographic factors.

Our results indicate that baseline functioning differs between treatment groups, most notably in relation to levels of sexual function. The baseline sexual function score for EBRT in our cohort was very low compared with other cohorts [3– 5]. This may be due to referral patterns with older more comorbid patients more likely to receive EBRT in our cohort. The largest change in function at 12 months, across all treatment groups, was seen for sexual functioning. Results of multivariable models confirmed RP’s greater impact on sexual function compared with other treatments. Unsurprisingly, the level of sexual bother was also much higher after RP compared with EBRT, brachytherapy and active surveillance, with 43% of RP patients reporting a moderate-to-big sexual problem after 12 months of treatment. This may be partly related to age, given the median age was much lower in the RP than the EBRT group, and younger men often have high sexual functioning expectations [21].

Psycho-social factors relating to having cancer which are known to affect men’s ability to achieve or maintain sexual activity, may be contributing to declined sexual function and increased sexual bother across all four treatment groups [22]. Providing sexual assessment, counselling, information, and emotional support following a diagnosis could improve sexual function and expectations among some men with prostate cancer. Interestingly, we found that men on active surveillance experienced a decline in sexual function 12 months after diagnosis, with an adjusted mean change score of − 22.1 points. More men on active surveillance were also bothered by their sexual dysfunction, with the proportion reporting sexual bother increasing from 20% at baseline to 37% at 12 months. Smith et al. (2009) reported a decline in sexual function in men on active surveillance compared to age-matched controls without prostate cancer [3]. Further studies are required to determine the extent to which men with low-risk prostate cancer experience sexual dysfunction while on active surveillance and to identify factors that may account for the decline.

Functional scores for urinary continence had also declined from relatively similar levels at baseline among all treatment groups. Results from adjusted models indicate that post-treatment urinary incontinence was worse after RP compared with the other three treatments. Conversely, men who received RP showed clinically significant positive change (improvement) in urinary irritative/obstructive symptoms after 12 months. This finding is in line with previous research [5, 7] in which authors hypothesised that, for men with coexisting hyperplasia of the prostate gland, removing the prostate improves urinary obstructive symptoms.

Our findings indicate that a clinically significant decline in bowel function was observed among men who had EBRT compared with RP. However, we did not observe any clinically meaningful change in bowel function 12 months after receiving brachytherapy. Bowel related complications from radiotherapy for prostate cancer have been observed previously [3, 5, 6], and are reportedly worse for EBRT compared with brachytherapy [23], though dose and technique can have a considerable impact [24].

Australia’s national prostate cancer registry, PCOR-ANZ, does not collect baseline PROMs, as is the case for other international prostate cancer registries [11]. Our models showed that baseline functional measures were positively associated with their respective 12 months functional measure across all domains, in line with previous studies [4, 5]. Our findings with respect to baseline measures provide a reference for future PCOR-ANZ studies reporting 12-month functional outcomes. Accurate assessment of baseline PROMs is required to properly estimate the harms attributed to prostate cancer treatment over time and failure to adjust for baseline differences could result in over-estimation of treatment-related harms and thereby erroneously shift the balance of benefits and harms [25]. In our study, without adjusting for baseline sexual function and other covariates, men who had received EBRT had lower scores in 12-months sexual function than RP (mean difference = − 9.7 points; 95% CI, − 13.6 to − 5.8) (Supplementary Table S5) but after adjusting for baseline characteristics, men in the EBRT group had higher scores (mean difference = 6.4 points; 95% CI, 0.9–12.0) in post-treatment sexual function compared with RP.

There have been advancements in radical therapy techniques that have led to an improvement in functional and oncologic outcomes. For example, different nerve-sparing surgical techniques [26–28] and more accurate delivery of radiotherapies such as Intensity-modulated Radiation Therapy (IMRT) and Image Guided Radiation Therapy (IGRT) has led to an improvement in functioning after treatment [29, 30]. The scope of our study was to compare the impacts between the main prostate cancer treatment groups not to compare specific treatment techniques.

The findings of this study should be interpreted in light of the following limitations. First, SA-PCCOC database captures the majority of prostate cancer cases in South Australia, and a significant proportion of men in the database had missing outcome responses for baseline and/or 12 months PROMs. We have used inverse probability weighting to minimise this bias. This cohort represents patients managed in both large tertiary centres (with associated government funded prostate cancer nurses) as well as single clinician private practices meaning that pre-treatment counselling may vary, which in turn may influence patient expectations, and thus, their responses. Second, our study did not assess further functionality changes overtime. While some studies showed that the changes over time (especially after 2 years) may be little and may not be significantly different between treatment categories [5, 6], other studies [31, 32] reported significant differences in functional outcome between treatments after 5 years. Third, the use of secondary treatment regimens (adjuvant or salvage therapy with radical therapies or conversion from active surveillance to treatment) was not addressed. The significant decline in post-treatment bowel and sexual function in the active surveillance group could be due to some men converting to radical therapies. Fourth, although active surveillance and watchful waiting are two different management options, some misclassification is likely within population-wide registries [11] which may have impacted findings with respect to the surveillance group. Also, our brachytherapy group included both those receiving high-dose and low-dose rate brachytherapy, with the majority (70%) having undergone low-dose rate brachytherapy. In South Australia, high-dose rate brachytherapy is generally combined with EBRT and hence likely to have a different side effect profile to that of low-dose rate brachytherapy. Moreover, the type of radical treatment technique was not separately compared which may have an effect in the level of function decline. Fifth, although the final models were adjusted for sociodemographic and baseline characteristics, there may be residual confounding due to unmeasured covariates, for example previous medical and surgical histories, which are likely to differ between treatment groups. Finally, while most of our findings are in line with previous population-based studies [3–6, 33, 34], the lack of consistency in the treatment approaches compared, PROMs measurement tools used, differences in follow-up periods, and the outcomes studied limits our ability to compare our results *directly* with other PROMs research [7, 8]. It should be highlighted that our findings are based on ‘real world data’, and as such, reflect the actual experiences of men during their prostate cancer journey.

## Conclusion

Baseline PROMs differed among men who underwent different prostate cancer management approaches, with the biggest difference in sexual function score. Clinically meaningful declines in sexual function were seen across all treatments 12 months post-treatment, along with declines in urinary continence following RP, and bowel function following EBRT. Whilst EBRT and active surveillance were associated with less 12 months post-treatment sexual bother than RP, bowel bother was greater for EBRT than RP. Assessment of baseline PROMs is important to appropriately estimate adverse effects of prostate cancer treatments on physical function and to provide appropriate counselling and support.

## Electronic supplementary material

Below is the link to the electronic supplementary material.

Supplementary Material 1

## Data Availability

The data that support the findings of this study are available from SA-PCCOC registry (https://www.prostatehealth.org.au/) but restrictions apply to the availability of these data, which were used under license for the current study, and so are not publicly available. Data are however available from the authors upon reasonable request and with permission of SA-PCCOC data custodians.

## List of abbreviations

EBRT: External beam radiation therapy.
EPIC: Expanded prostate cancer index composite.
MCID: Minimal clinically important difference.
PCOR-ANZ: Prostate cancer outcomes registry Australia and New Zealand.
RP: Radical prostatectomy.
SA-PCCOC: South Australian prostate cancer clinical outcomes collaborative.
SEIFA: Socio-economic indexes for areas.
