# Supplementary material for: Patient-reported functional outcome measures and treatment choice for prostate cancer
Source: BMC Urol. 2022 Nov 5;22:169. doi: 10.1186/s12894-022-01117-1 (PMC9637295; doi:10.1186/s12894-022-01117-1)
Supplement: Supplementary file 1 — Supplementary Material 1 [file 12894_2022_1117_MOESM1_ESM.docx]

Table S1: Minimally clinically important difference (MCID) scores (based on change scores from baseline to 12 months) by treatment type, SA-PCCOC registry (2010 – 2019)

| **Functional domain** | **Adjusted**‡ | | | | **Standard cut-off^#^** |
| --- | --- | --- | --- | --- | --- |
|  | **Radical Prostatectomy** | **External Beam Radiation Therapy** | **Brachytherapy** | **Active Surveillance** |  |
| Sexual function | –28.9* | –16.5* | –17.8* | –22.1* | 10-12 |
| Urinary incontinence | –15.6* | –2.2 | –5.4 | –5.9 | 6-9 |
| Urinary obstruction | 5.6* | 2.0 | 1.7 | 3.9 | 5-7 |
| Bowel function | –0.8 | –6.3* | –1.1 | –4.4* | 4-6 |

^#^Skolarus et al, 2014.

‡Adjusted for age, SEIFA score, PSA level, risk category and primary symptoms.

The lower value was selected to indicate MCID in the current study.

*MCID observed in: urinary incontinence and obstruction in RP, bowel function in EBRT, bowel function in AS, and sexual function in all treatments.

Table S2: Unadjusted (crude) patient reported functional outcome scores at baseline and 12 months after treatment, SA-PCCOC registry (2010 – 2019)

|  |  | **Radical Prostatectomy** | | | **External Beam Radiation Therapy** | | | **Brachytherapy** | | | **Active Surveillance** | | |  |
| --- | --- | --- | --- | --- | --- | --- | --- | --- | --- | --- | --- | --- | --- | --- |
| **Baseline scores** | **Functional domain** | **No.** | **p50** | **iqr** | **No.** | **p50** | **iqr** | **No.** | **p50** | **iqr** | **No.** | **p50** | **iqr** | **p^¥^** |
|  | Sexual function | 1667 | 70.8 | 36.2–87.5 | 312 | 26.6 | 12.5–58.3 | 96 | 77.8 | 48.7–87.5 | 324 | 62.5 | 32–87.5 | <0.001 |
|  | Urinary incontinence | 1797 | 100 | 87.5–100 | 392 | 100 | 79.3–100 | 119 | 100 | 85.5–100 | 359 | 100 | 79.3–100 | <0.001 |
|  | Urinary obstruction | 1803 | 93.8 | 81.3–100 | 399 | 87.5 | 75–93.8 | 120 | 87.5 | 75–100 | 358 | 87.5 | 75–100 | <0.001 |
|  | Bowel function | 1834 | 100 | 91.7–100 | 427 | 95.8 | 87.5–100 | 123 | 95.8 | 83.3–100 | 372 | 95.8 | 87.5–100 | <0.001 |
|  |  |  |  |  |  |  |  |  |  |  |  |  |  |  |
| **12 months scores** | **Functional domain** | **No.** | **p50** | **iqr** | **No.** | **p50** | **iqr** | **No.** | **p50** | **iqr** | **No.** | **p50** | **iqr** | **p^¥^** |
|  | Sexual function | 1207 | 18.0 | 8.3–48.7 | 184 | 16.7 | 4.6–24.3 | 83 | 45.8 | 16.7–66.7 | 163 | 41.7 | 13.8–75 | <0.001 |
|  | Urinary incontinence | 1381 | 79.3 | 58.5–100 | 241 | 93.8 | 75–100 | 113 | 100 | 85.5–100 | 195 | 91.8 | 71–100 | <0.001 |
|  | Urinary obstruction | 1376 | 93.8 | 87.5–100 | 238 | 87.5 | 75–93.8 | 113 | 87.5 | 75–93.8 | 188 | 93.8 | 81.3–100 | <0.001 |
|  | Bowel function | 1416 | 100 | 91.7–100 | 259 | 87.5 | 75–95.8 | 119 | 95.8 | 83.3–100 | 200 | 100 | 87.5–100 | <0.001 |
|  |  |  |  |  |  |  |  |  |  |  |  |  |  |  |
| **Change scores** | **Functional domain** | **No.** | **Mean** | **sd** | **No.** | **Mean** | **sd** | **No.** | **Mean** | **sd** | **No.** | **Mean** | **sd** | **p^‡^** |
|  | Sexual function | 806 | –30.6* | 29.6 | 62 | –15.2* | 25.2 | 33 | –16.1* | 25.4 | 96 | –18.7* | 27 | <0.001 |
|  | Urinary incontinence | 949 | –15.8* | 25.3 | 100 | –4.0 | 17.4 | 51 | –4.3 | 16.5 | 122 | –5.1 | 19.5 | <0.001 |
|  | Urinary obstruction | 967 | 5.8* | 15.7 | 96 | 2.9 | 18.5 | 51 | 1.8 | 19.5 | 117 | 3.4 | 13.8 | 0.130 |
|  | Bowel function | 999 | –0.9 | 12.6 | 118 | –8.3* | 21 | 59 | 0.2 | 12.6 | 120 | –1.0 | 9.2 | <0.001 |

- p50, median score; iqr, inter quartile range; sd, standard deviation.
- p^¥^, p-values are based on Kruskal-Wallis H test; p**^‡^**, p-values are based on one-way ANOVA.
- For each functional domain, the EPIC scores range from 0 (worst) to 100 (best).
- *MCID observed in: urinary incontinence and obstruction in RP, bowel function in EBRT, and sexual function in all treatments.

Table S3: Adjusted regression outputs of the effect of RP, EBRT, brachytherapy *vs.* active surveillance on 12-month post-treatment functional outcome scores and bothers, SA-PCCOC registry (2010 – 2019)

| **Outcome measures** | **Active Surveillance** | **Radical Prostatectomy** | | **External Beam Radiation Therapy** | | **Brachytherapy** | |
| --- | --- | --- | --- | --- | --- | --- | --- |
|  |  | Mean difference^‡^ | 95% CI | Mean difference^‡^ | 95% CI | Mean difference^‡^ | 95% CI |
| Sexual function | reference | –6.9 | –14.3, 0.5 | –0.5 | –8.9, 8.0 | 10.5 | 0.3, 20.7* |
| Urinary incontinence | reference | –10.6 | –15.3, –5.9*** | 3.0 | –3.1, 9.0 | 0.1 | –7.6, 7.6 |
| Urinary obstruction | reference | 0.2 | –3.1, 3.5 | –7.0 | –11.6, –2.5** | –6.8 | –12.2, –1.3* |
| Bowel Function | reference | 2.1 | –0.2, 4.4 | –5.8 | –10.7, –1.0* | 0.3 | –3.6, 4.1 |
|  |  |  |  |  |  |  |  |
|  |  | Adjusted OR‡‡ | 95% CI | Adjusted OR‡‡ | 95% CI | Adjusted OR‡‡ | 95% CI |
| Sexual bother | reference | 1.65 | 1.04, 2.63* | 0.84 | 0.41, 1.73 | 1.12 | 0.56, 2.24 |
| Urinary bother | reference | 1.56 | 0.98, 2.50 | 1.17 | 0.65, 2.11 | 1.19 | 0.56, 2.52 |
| Bowel bother^¥^ | reference | 0.86 | 0.50, 1.50 | 1.73 | 0.87, 3.45 | - | - |

- CI, confidence interval
- ^‡^the mean difference in the 12 months functional scores between RP, EBRT, brachytherapy vs. active surveillance from linear regression models adjusted for baseline score of the respective function, age, SEIFA score, PSA level, risk category and primary symptoms.
- ‡‡OR, odds ratio from ordinal logistic regression models of 12-month post-treatment bothers (five categories: no, very small, small, moderate and big bothers) adjusted for baseline bother of the respective function, age, SEIFA score, PSA level, risk category and primary symptoms.
- All analyses were with the weighted sample.
- ^¥^The brachytherapy group was not included in the model because there were very small counts in brachytherapy group.
- *p<0.05, **p<0.01, ***p<0.001.

Table S4: Extent of bother at baseline and 12 months across each treatment category, SA-PCCOC registry (2010 – 2019)

| **Bother type** |  | **Baseline** | | | | | | | | | | | **12 months** | | | | | | | | | | |
| --- | --- | --- | --- | --- | --- | --- | --- | --- | --- | --- | --- | --- | --- | --- | --- | --- | --- | --- | --- | --- | --- | --- | --- |
|  |  | No | | Very small | | Small | | Moderate | | Big | | p^¥¥^ | No | | Very small | | Small | | Moderate | | Big | | p^¥¥^ |
|  |  | No. | % | No. | % | No. | % | No. | % | No. | % |  | No. | % | No. | % | No. | % | No. | % | No. | % |  |
| Sexual bother | RP | 753 | 44.6 | 336 | 19.9 | 272 | 16.1 | 183 | 10.8 | 143 | 8.5 | <0.001 | 226 | 19.0 | 202 | 17.0 | 232 | 19.5 | 248 | 20.8 | 282 | 23.7 | 0.001 |
|  | EBRT | 81 | 25.6 | 85 | 26.9 | 58 | 18.4 | 45 | 14.2 | 47 | 14.9 |  | 61 | 31.4 | 30 | 15.5 | 26 | 13.4 | 33 | 17.0 | 44 | 22.7 |  |
|  | BT | 41 | 42.3 | 25 | 25.8 | 15 | 15.5 | 9 | 9.3 | 7 | 7.2 |  | 18 | 22.0 | 16 | 19.5 | 18 | 22.0 | 19 | 23.2 | 11 | 13.4 |  |
|  | AS | 118 | 36.0 | 69 | 21.0 | 65 | 19.8 | 45 | 13.7 | 31 | 9.5 |  | 45 | 26.8 | 39 | 23.2 | 22 | 13.1 | 33 | 19.6 | 29 | 17.3 |  |
|  | Overall* | 1108 | 39.6 | 601 | 21.5 | 474 | 16.9 | 334 | 11.9 | 281 | 10.0 |  | 398 | 22.6 | 308 | 17.5 | 321 | 18.2 | 346 | 19.6 | 391 | 22.2 |  |
|  |  |  |  |  |  |  |  |  |  |  |  |  |  |  |  |  |  |  |  |  |  |  |  |
| Urinary bother | RP | 965 | 51.4 | 497 | 26.5 | 235 | 12.5 | 142 | 7.6 | 37 | 2.0 | <0.001**^‡‡^** | 612 | 42.9 | 485 | 34.0 | 192 | 13.4 | 96 | 6.7 | 43 | 3.0 | 0.567 |
|  | EBRT | 141 | 32.6 | 145 | 33.6 | 79 | 18.3 | 52 | 12.0 | 15 | 3.5 |  | 108 | 39.7 | 86 | 31.6 | 50 | 18.4 | 21 | 7.7 | 7 | 2.6 |  |
|  | BT | 57 | 46.3 | 30 | 24.4 | 22 | 17.9 | 13 | 10.6 | 1 | 0.8 |  | 52 | 44.1 | 41 | 34.7 | 14 | 11.9 | 8 | 6.8 | 3 | 2.5 |  |
|  | AS | 170 | 44.5 | 109 | 28.5 | 56 | 14.7 | 30 | 7.9 | 17 | 4.5 |  | 94 | 46.5 | 67 | 33.2 | 21 | 10.4 | 17 | 8.4 | 3 | 1.5 |  |
|  | Overall* | 1492 | 44.7 | 945 | 28.3 | 503 | 15.1 | 299 | 9.0 | 101 | 3.0 |  | 944 | 42.8 | 737 | 33.4 | 304 | 13.8 | 154 | 7.0 | 65 | 3.0 |  |
|  |  |  |  |  |  |  |  |  |  |  |  |  |  |  |  |  |  |  |  |  |  |  |  |
| Bowel bother | RP | 1315 | 70.2 | 388 | 20.7 | 106 | 5.7 | 44 | 2.4 | 19 | 1.0 | <0.001**^‡‡^** | 948 | 66.2 | 315 | 22.0 | 106 | 7.4 | 46 | 3.2 | 18 | 1.3 | <0.001**^‡‡^** |
|  | EBRT | 188 | 42.9 | 162 | 37.0 | 54 | 12.3 | 24 | 5.5 | 10 | 2.3 |  | 103 | 38.1 | 96 | 35.6 | 36 | 13.3 | 28 | 10.4 | 7 | 2.6 |  |
|  | BT | 77 | 61.6 | 28 | 22.4 | 14 | 11.2 | 6 | 4.8 | 0 | 0.0 |  | 75 | 64.1 | 28 | 23.9 | 13 | 11.1 | 1 | 0.9 | 0 | 0.0 |  |
|  | AS | 243 | 63.6 | 85 | 22.3 | 34 | 8.9 | 14 | 3.7 | 6 | 1.6 |  | 136 | 67.7 | 41 | 20.4 | 11 | 5.5 | 10 | 5.0 | 3 | 1.5 |  |
|  | Overall* | 2070 | 62.0 | 815 | 24.4 | 268 | 8.0 | 134 | 4.0 | 53 | 1.6 |  | 1371 | 62.1 | 534 | 24.2 | 181 | 8.2 | 93 | 4.2 | 30 | 1.4 |  |

RP, radical prostatectomy; EBRT, external beam radiation therapy; BT, brachytherapy; AS, active surveillance.

*, Overall bother score; p^¥¥^, p-values are based on Pearson’s chi-square; **^‡‡^**p-values are based on Fisher's exact test.

All the scores are unadjusted.

Table S5: Crude and adjusted regression outputs of the effect of EBRT, brachytherapy, active surveillance vs. RP on 12-month post-treatment functional outcome scores and bothers, SA-PCCOC registry (2010 – 2019)

| **Outcome measures** | **RP** | **External Beam Radiation Therapy** | | | | **Brachytherapy** | | | | **Active Surveillance** | | | |
| --- | --- | --- | --- | --- | --- | --- | --- | --- | --- | --- | --- | --- | --- |
|  |  | **Crude** | | **Adjusted** ^‡^ | | **Crude** | | **Adjusted** ^‡^ | | **Crude** | | **Adjusted** ^‡^ | |
|  |  | $\beta$ | 95% CI | $\beta$ | 95% CI | $\beta$ | 95% CI | $\beta$ | 95% CI | $\beta$ | 95% CI | $\beta$ | 95% CI |
| Sexual function | reference | –9.68 | –13.55, –5.81*** | 6.44 | 0.91, 11.97* | 15.93 | 8.25, 23.61*** | 17.43 | 9.4, 25.46*** | 11.59 | 5.83, 17.35*** | 6.92 | –0.49, 14.34 |
| Urinary incontinence | reference | 12.58 | 9.55, 15.62*** | 13.59 | 8.98, 18.20*** | 13.35 | 8.81, 17.88*** | 10.61 | 3.93, 17.29*** | 9.87 | 6.16, 13.58*** | 10.60 | 5.93, 15.28*** |
| Urinary obstruction | reference | –7.63 | –10.02, –5.24*** | -7.23 | –10.70, –3.75*** | –9.02 | –12.49, –5.56*** | -6.94 | –11.68, –2.21*** | –3.67 | –6.71, –0.61* | -0.19 | –3.48, 3.11 |
| Bowel Function | reference | –9.62 | –12.27, –6.96*** | –7.93 | –12.39, –3.46*** | –0.97 | –3.49, 1.56 | –1.84 | –5.14, 1.47 | –1.26 | –3.89, 1.37 | –2.09 | –4.37, 0.19 |
|  |  |  |  |  |  |  |  |  |  |  |  |  |  |
|  |  | **OR** | **95% CI** | **OR** | **95% CI** | **OR** | **95% CI** | **OR** | **95% CI** | **OR** | **95% CI** | **OR** | **95% CI** |
| Sexual bother | reference | 0.63 | 0.44, 0.89** | 0.51 | 0.29, 0.89* | 0.76 | 0.50, 1.15 | 0.68 | 0.36, 1.27 | 0.70 | 0.51, 0.97* | 0.60 | 0.38, 0.96* |
| Urinary bother | reference | 1.12 | 0.85, 1.49 | 0.75 | 0.49, 1.15 | 0.91 | 0.62, 1.32 | 0.76 | 0.41, 1.41 | 0.63 | 0.45, 0.88** | 0.64 | 0.40, 1.03 |
| Bowel bother* | reference | 2.13 | 1.67, 2.71*** | 1.66 | 1.13, 2.45** | - | - | - |  | 0.93 | 0.64, 1.35 | 1.11 | 0.64, 1.92 |
|  |  |  |  |  |  |  |  |  |  |  |  |  |  |

- RP, radical prostatectomy; CI, confidence interval; β denotes the mean difference in the 12 months functional scores between EBRT, brachytherapy, active surveillance vs. RP from linear regression models.
- ^‡^The models were adjusted for inverse probability weight, baseline score of the respective function, age, SEIFA score, PSA level, risk category and primary symptoms.
- OR, odds ratio from ordinal logistic regression models of 12-month post-treatment bothers (five categories: no, very small, small, moderate and big bothers).

All analyses were with the weighted sample.

- ^¥^The two groups of radiotherapy (EBRT and brachytherapy) were merged because there were very small counts in brachytherapy group.
- *p<0.05, **p<0.01, ***p<0.001.
